# Diversity of Microbial Communities of *Pinus sylvestris* var. *mongolica* at Spatial Scale

**DOI:** 10.3390/microorganisms10020371

**Published:** 2022-02-05

**Authors:** Dan-Dan Wang, Wen Zhao, Mumin Reyila, Kai-Chuan Huang, Shun Liu, Bao-Kai Cui

**Affiliations:** Institute of Microbiology, School of Ecology and Nature Conservation, Beijing Forestry University, Beijing 100083, China; wdd1360303992@163.com (D.-D.W.); zhaowendrlw@163.com (W.Z.); Ramilla@163.com (M.R.); Huangkc1014@bjfu.edu.cn (K.-C.H.); liushun2017@bjfu.edu.cn (S.L.)

**Keywords:** soil microbial diversity, *Pinus sylvestris* var. *mongolica*, spatial scale, soil properties

## Abstract

Soil microorganisms play an indispensable role in the forest ecosystem. It is necessary to study the soil microorganisms in *Pinus sylvestris* var. *mongolica*, which is one of the afforestation species widely planted in the northern sandy region of China. We collected soil samples of *P. sylvestris* at large spatial scales and analyzed bacterial and fungal community composition differences using high-throughput sequencing techniques. The results showed that: (1) the richness index of different sandy lands was significantly different. The α-diversity of bacteria was the highest in Mu Us Sandy Land, and the α-diversity of fungi was the highest in Horqin Sandy Land. (2) The dominant phyla of bacteria were Actinobacteria, Proteobacteria, Chloroflexi and Acidobacteria, while the dominant phyla of fungi were Ascomycota and Basidiomycota. The relative abundance of dominant phyla was different. (3) Temperature and precipitation were the main driving factors of bacterial and fungal community change at large spatial scale. In addition, bacteria were also affected by total nitrogen, soil organic carbon and pH content; fungal community was affected by pH. The microorganisms showed obvious differences in geographical distribution, which could provide ideas for promoting sustainable management of *P. sylvestris* stand.

## 1. Introduction

Soil microorganism: a general term for microscopic organisms living in soil that are indistinguishable from the naked eye. It is estimated that one gram of soil contains up to hundreds of millions of microorganisms [1], among which bacteria and fungi are the key organisms, given their great diversity and richness. It has been dubbed “microbial dark matter” due to its vast variety and unknown function [2]. The high heterogeneity of the soil environment, which is an important hub connecting various spheres and ecosystems, breeds a great diversity of microorganisms; it is also the engine of the soil element cycle and maintains the sustainable development of human beings and the Earth’s ecosystem [3,4]. Soil microorganisms play an indispensable role in the forest ecosystem [5,6]. It is the bridge between aboveground vegetation and the underground soil system [7,8], participating in carbon and nitrogen cycles, regulating the decomposition of organic matter and energy flow [9,10,11,12,13,14], and affecting the succession of plant community structure [8]. The sensitive response of microbial diversity to the surrounding environment reflects the stability and function of an ecosystem to some extent and can be an important indicator of soil change [15].

The diversity and composition of microbial communities show great spatial variability and are associated with changes in some biological or abiotic factors [16,17,18]. Above-ground vegetation provides nutrients needed for the survival of soil microorganisms in the process of material exchange with the external environment and changes the composition of the microbial community by affecting soil properties through litter [19] and root exudates [20,21]. The complex environmental characteristics of soil and geographic factors play a prominent role in determining the soil microbial community structure [22]. There is no conclusion that soil pH has a significant impact on microbial diversity. Some previous studies have described a strong influence of pH on the composition of microbial communities over different scales. Bacterial community structure changes significantly even in a very small variation of pH values [23,24,25]. Fierer and Jackson [26] and Griffiths et al. [27] have demonstrated that pH is a primary factor in a continent-wide survey of microbial communities; other studies have shown that pH has nothing to do with bacterial community structure [28]. There is a coevolution between plants and microorganisms. Plants provide nutrients for microorganisms, and microorganisms transform litter into inorganic nutrients needed for plant growth through decomposition. Rhizosphere nutrients and bioavailability directly or indirectly effect the microbiome assembly and functions [29]. Banerjee et al. [30] found that soil moisture effected microbial community structure more than nutrients. Similarly, the influence of geographical factors and climatic factors, such as temperature, altitude and precipitation on soil microbial diversity, has not been determined [31,32,33].

*Pinus sylvestris* var. *mongolica* (*P. sylvestris* for short below), a geographical variety species of the Scots pine, is the widely planted arbor for constructing protective plantations in “Three North” (Northwest, North and Northeast of China) [34,35], on account of its fast growth and strong adaptability to poor environmental conditions. Up until now, most of the studies on *P. sylvestris* have focused on the diversity of fungi or ectomycorrhizal fungi at a particular location, the change of fungal community with a shift ingrowth stage or the microbial difference between plantation and natural forest and have ignored understanding the microbial community assembly across spatial scales.

In this study, we collected soil of *P. sylvestris* from different areas and studied the community structure of bacteria and fungi, in order to provide some basis for solving the decline phenomenon of *P. sylvestris* in introduced areas. High-throughput sequencing technology was applied to study the composition of the microbial community. To some extent, the composition of microbial communities in different introduced areas reflects the preference of their respective environments. We attempted to explore the response of microorganisms to environmental factors at a large spatial scale, which helped to clarify the reasons for the decline of *P. sylvestris* in the introduction area, providing some theoretical support for the introduction of the *P. sylvestris* plantation. We hypothesized that (1) microbial diversity was different in different locations and that the diversity of a healthy sand was higher than that of declining area; (2) the composition of microbial community was more sensitive to climatic factors than soil factors.

## 2. Materials and Methods

### 2.1. Site Information

**Figure 1:**
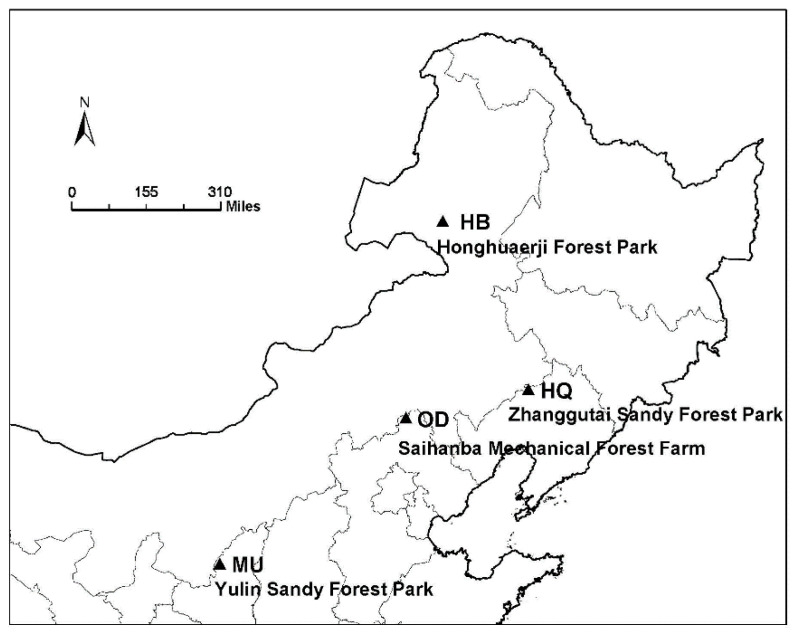
Sampling locations. HB: the Hulunbuir Sandy Land; OD: the Otindag Sandy Land; MU: the Mu Us Sandy Land; HQ: the Horqin Sandy Land.

We conducted this study in four typical areas of *P. sylvestris* forest (Figure 1). Specific site information is shown in the Table 1.

Honghuaerji Forest Park (Inner Mongolia Autonomous Region), located in the southeast of Hulunbuir (HB) Sandy Land, with a sandy *P. sylvestris* system and a grassland wetland landscape, is at an elevation of 500–1000 m, with a temperate continental monsoon climate, characterized by rain and heat in the same season [36]. Beautiful mountains and rivers, rich flora and fauna make it a local tourist hotspot.

Saihanba Mechanical Forest Farm (Hebei Province), located in the southern edge of Otindag (OD) Sandy Land, is at an elevation of about 1510–1939 m, with a cold temperature continental monsoon climate, with long winters and low temperatures [37]. The forest coverage rate has been greatly improved by artificial afforestation and ecological management in the past 60 years. There are vast forests and grasslands, clear plateau lakes and historical relics of the Qing Dynasty. Manchu and Mongolian people have lived here for a long time, and their national cultures blend with each other, giving birth to rich folk customs. Its unique geographical location breeds abundant animal and plant resources, as well as natural hydrological resources and cultural attractions, which bring it unique charm.

Yulin Sandy Forest Park (Shaanxi Province), located in the southern edge of Mu Us (MU) Sandy Land, is at an elevation of about 1120 m, with a semi-arid continental monsoon climate. Its annual precipitation rate varies greatly; there are more rainstorms in the summer, adequate light and heat which are sufficient for plant growth, and large temperature differences between day and night [38]. The sandy land was seriously eroded by wind and sand at first but now, the forest coverage rate has been significantly improved by ecological management; the cultural landscape and tourism industry have been developed.

Zhanggutai Sandy Forest Park (Liaoning Province), located in the southern margin of Horqin (HQ) Sandy Land, is at an elevation of 220–240 m, with a temperate continental monsoon climate, characterized by simultaneous humidity and heat, and little precipitation, mainly concentrated in the summer [39]. It is an ecological landscape combining desert wonders and artificial forests. The reserves of silica sand and peat are abundant; the seedlings of *P. sylvestris* have been cultivated for several decades.

### 2.2. Soil Sampling

Experimental samples were collected in April–May 2021; plantations of two sampling sites in each test location were selected for study; three 20 × 20 m^2^ plots were taken from each place as repeated groups. *P. sylvestris* soil samples of 0–20 cm layer collected with a soil drill were pooled into sealed bags; stones, herbs roots, and litters were excluded with 2 mm mesh [40]. The plots were separated by more than 100 m. A five-point sampling method was adopted, with three repeated soil samples collected in each point. All the composite samples (4 sandy lands × 2 places × 3 × 5 = 120 composite samples) were stored in dry ice for transport to the lab for analysis.

### 2.3. Environmental Variables

The soil samples were divided into two parts: one was naturally air-dried for the determination of soil physical and chemical properties; the other was used for high-throughput sequencing of soil microorganisms.

For the determination of soil properties, we referred to the method of Bao [41]. The soil pH value was obtained by measuring the suspension with a soil–water ratio of 1:2.5 using a pH meter; soil organic carbon (SOC) was determined by titration of ferrous sulfate heated by potassium dichromate; total nitrogen (TN) was determined by using the Kelvin method, with Se, CuSO_4_, and K_2_SO_4_ as catalysts, adding concentrated sulfuric acid digestion, with 1 g of soil. MADAC (Anti-molybdenum antimony colorimetry) was used to estimate the phosphorus content in samples from soil. The determination of cation exchange capacity (CEC) was determined by ammonium acetate exchange using the Kjeldahl method.

Climate data for each sampling location were obtained from the China Meteorological Data Service Center (CMDC, http://data.cma.cn/en; accessed on 15 July 2021), including the mean monthly temperature (Ta), mean monthly precipitation (Pa), monthly average maximum and minimum temperature (T+ and T−).

### 2.4. Soil DNA Extraction and Amplification

Total genomic DNA were extracted using the DNeasy Power Soil Pro Kit (QIAGEN, Frankfurt, Germany) and stored at −20 °C prior to further analysis. A NanoDrop NC2000 spectrophotometer (Thermo Fisher Scientific, Waltham, MA, USA) was used to quantify DNA; the quality of DNA was detected by 1.2% agarose gel electrophoresis. For PCR amplification of the bacterial 16S rRNA genes at the V3–V4 region, the primers were 338F (5′-ACTCCTACGGGAGGCAGCA-3′) and 806R (5′-GGACTACHVGGGTWTCTAAT-3′) [42]. For the ITS1 regions of fungi, the primers were ITS1F (5-GGAAGTAAAAGTCGTAACAAGG-3) and ITS2 (5-TCCTCCGCTTATTGATATGC-3) [43]. The PCR amplification was performed by Applied Biosystems 2720 Thermal Cycler (Thermo Fisher Scientific, Waltham, MA, USA); the Quant-iT PicoGreen dsDNA assay was performed by a Microplate Reader FLx800 (BioTek, Burlington, VT, USA). PCR amplicons were purified and recovered by adding Vazyme VAHTSTM DNA Clean Beads (Vazyme, Nanjing, China) and quantified with the fluorescent reagent of Quant-iT PicoGreen dsDNA assay kit (Invitrogen, Carlsbad, CA, USA); then, amplicons were mixed in proportion to the sequencing amount, and pair-end 2 × 250 bp sequencing was performed using the Illlumina MiSeq platform with a NovaSeq 6000 SP reagent kit at Shanghai Personal Biotechnology Co., Ltd. (Shanghai, China).

### 2.5. Sequence Analysis

Microbiome bioinformatics were processed using QIIME2 2020.2 with slight modification according to the official tutorials (https://docs.qiime2.org/2020.11/tutorials/; accessed on 27 June 2021). Briefly, a Demux plugin was used for the sample splitting of mixed sequences and a Cutadapt plugin was used to cut primers [44]. Then, a DADA2 plugin was used for quality filtering, denoising and chimera removal of the sequences. [45]. After quality screening, non-singleton amplicon sequence variants (ASVs) were aligned with mafft and used to construct a phylogeny with fasttree2 [46,47]. The taxonomic assignment of the sequences was determined based on the bacterial SILVA reference database for 16S rRNA and the fungal UNITE reference database for ITS. Raw sequencing data have been submitted to the NCBI database (accession number: PRJNA785088).

### 2.6. Statistical Analysis

Sequence data analyses were mainly performed using QIIME2 and R packages (version 4.0.3). Microbial alpha diversity indices at the ASV level, including the Shannon diversity index, the Chao1 richness estimator, and Pielou’s evenness were calculated using the ASV number in QIIME2, and visualized as box plots. Under the condition that the test data meet the homogeneity of variance, one-way analysis of variance (ANOVA) was conducted to compare the soil properties, with *p* < 0.05 considered statistically significant, supplemented by post hoc Tukey’s tests. SPSS version 23.0 software (SPSS Inc., Chicago, IL, USA) was used for the analysis of variance. Non-metric multidimensional scaling (NMDS) was performed to test for differences in microbial communities in different locations based on unweighted UniFrac distance. Redundancy analysis (RDA), canonical correlation analysis (CCA) and Mantel tests were used to explain the relationship between the environmental factors and microbial composition. NMDS, RDA, CCA, and Mantel tests were performed in R.

## 3. Results

### 3.1. Soil Properties

The soil nutrient content in HB Sandy Land and OD Sandy Land was significantly higher than that in HQ Sandy Land and MU Sandy Land. The maximum value of TN, AP and CEC was found in HB Sandy Land; the maximum value of SOC was found in OD Sandy Land, the minimum value of TN, SOC was found in MU Sandy Land, and the minimum value of AP and CEC was found in HQ Sandy Land. The soils of MU Sandy Land were alkaline compared to the acidic soils of other sandy lands (Table 2).

### 3.2. Diversity of Bacterial and Fungal Communities

A total of 4,684,298 high-quality bacterial sequences were obtained from the samples; the sequences were grouped into 6730 ASVs. A total of 8,927,828 high-quality fungal sequences were grouped into 3687 ASVs.

**Figure 2:**
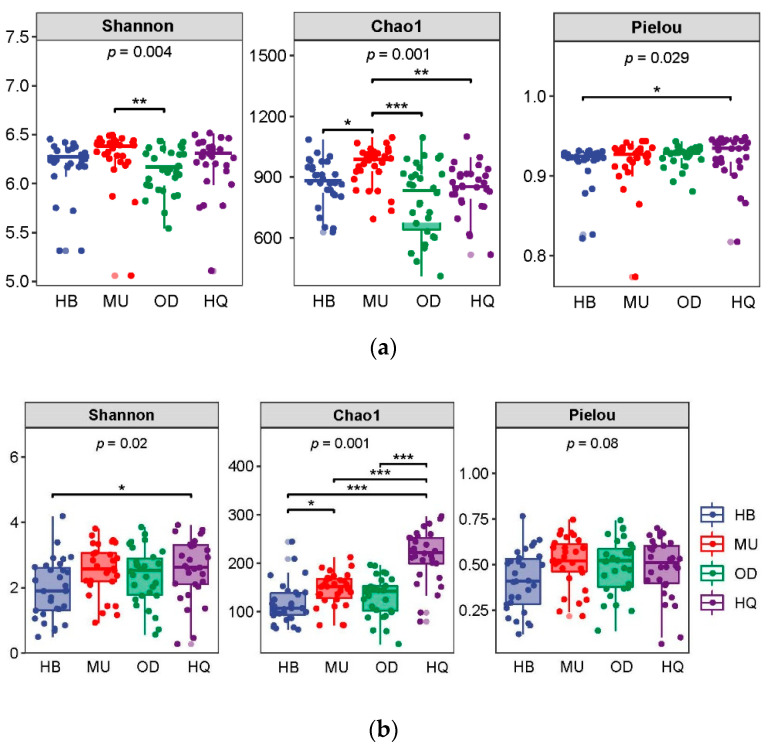
α diversity of soil bacterial (**a**) and fungal (**b**) communities in four sandy lands. (* *p* < 0.05; ** *p* < 0.01; *** *p* < 0.001).

For bacteria, diversity indices based on identified ASVs (Figure 2) showed that the diversity and richness indices at MU possessed the highest values, while the lowest values were found at OD. The Shannon index has a certain difference between MU and OD; the Pielou index showed a certain difference between HB and HQ. The Chao1 indices measurements differed significantly between MU and other locations (*p* < 0.05).

For fungi, the diversity and richness indices at HQ showed the highest values, while the lowest values were found at HB. The Shannon index possesses a certain difference between HB and HQ. The Chao1 indices measurements differed significantly between HQ and other locations (*p* < 0.05). There was no significant difference in the Pielou index of different sandy lands.

### 3.3. Bacterial and Fungal Community Composition

**Figure 3:**
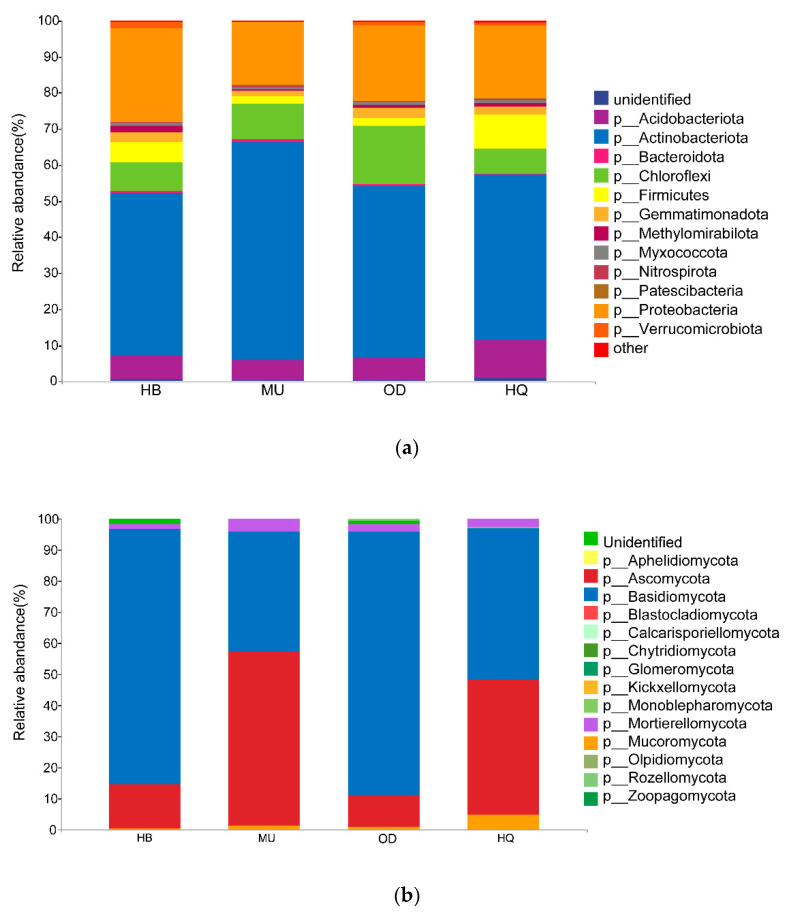
Relative abundances of bacterial (**a**) and fungal (**b**) phyla in each *P. sylvestris* stand across four sandy lands.

The relative abundances were calculated on the ASVs numbers (Figure 3).

In the composition of the bacterial community, a total of 25 phyla were detected. There were six dominant bacterial phyla with relative abundance that reached more than 1%, Actinobacteriota was the most abundant phylum (49.88%), followed by Proteobacteria (21.15%), and then Chloroflexi (10.23%), Acidobacteriota (7.18%), Firmicutes (4.75%), and Gemmatimonadota (2.31%). The bacterial community composition differed among test locations. Dadabacteria and Bdellovibrionota were only detected in the HQ Sandy Land, Armatimonadota, p_GAL15 and p_MBNT15 were only detected in the MU Sandy Land, Elusimicrobiota were absent in the OD Sandy Land and HQ Sandy Land.

For fungi, Basidiomycota, Ascomycota, Mortierellomycota, Mucoromycota, Rozellomycota and Zoopagomycota were found in each sampling site at the phylum level. There were four dominant phyla with relative abundance greater than 1%, Basidiomycota was the most abundant phylum (64.95%), immediately followed by Ascomycota (29.45%), and then Mortierellomycota (2.70%), in addition to the unidentified fungi group (1.83%).

**Figure 4:**
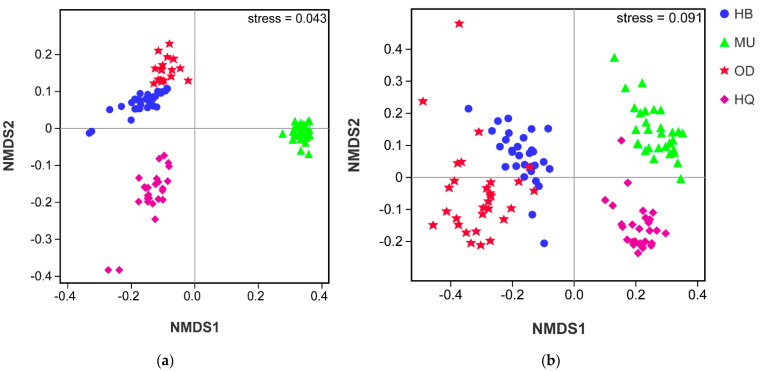
Bacterial (**a**) and fungal (**b**) community structure based on unweighted UniFrac distance, as determined by non-metric multidimensional scaling (NMDS).

Soil bacterial and fungal community structure with the different sandy land was analyzed using NMDS based on the unweighted UniFrac distance. NMDS analysis showed clear area separation in both bacteria and fungi (Figure 4). ANOSIM agreed with the NMDS in that sampling location caused differences in bacterial and fungal community structure (r = 0.951, *p* = 0.001 and r = 0.963, *p* = 0.001, respectively).

### 3.4. The Response of Bacterial and Fungal Communities to Environmental Variation

Shannon diversity, Chao1 richness and Pielou evenness indices of bacteria significantly correlated with AP and pH (Table 3). The most significant factors associated with the diversity index were soil physical and chemical properties and T− and Ta (*p* < 0.05). The Chao1 index was significantly correlated with all soil physical and chemical factors, except CEC. Only the Shannon index showed a certain correlation with meteorological data. 

For fungi, the Shannon index was significantly correlated with the AP, pH, and CEC (Table 4). The Pielou index was significantly correlated with all soil physical and chemical factors, except pH. The factors that were most significantly associated with the Shannon index included AP, pH and CEC. The only factor that affected the Chao1 index was pH.

**Figure 5:**
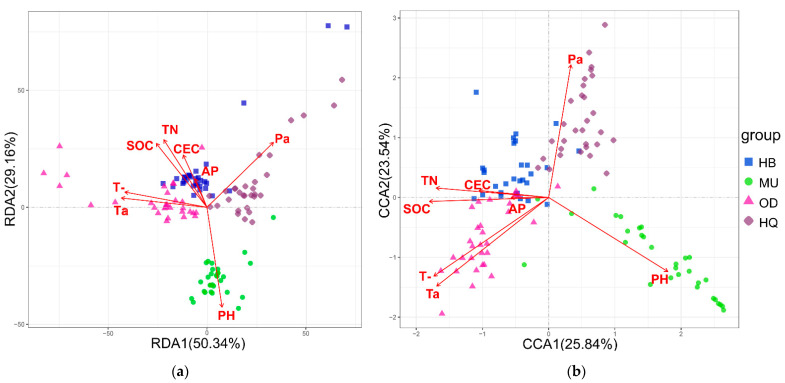
RDA of soil bacterial (**a**) and CCA of fungal (**b**) communities at the genus level and environmental variables.

To evaluate the relationship of microbial community structure with environmental variables at spatial scales, RDA analysis of bacteria, CCA analysis of fungal community and the Mantel test were implemented, according to the results of the DCA analysis. The results showed that the first two axes explained 79.50% and 49.38% of the total variation in bacteria and fungi, respectively (Figure 5). 

Based on the Mantel test, climate factor was remarkably correlated with bacterial (Table 5) and fungal (Table 6) community structure. The main factors that affected the bacterial community of soil were TN, SOC and pH. For the fungal community, pH showed a relatively obvious effect compared to other soil factors (r = 0.081, *p* = 0.018). The difference between MU Sandy Land in bacterial and fungal community and the other locations was caused by the pH. Pa was the main factor that explained the differences in community composition between HQ Sandy Land and others.

## 4. Discussion

### 4.1. Variation in Bacterial and Fungal Composition

The differences in microbial diversity are mainly in the richness index. The bacterial Chao1 richness index in MU Sandy Land was significantly higher than other locations, and the fungal Chao1 richness index in HQ Sandy Land was significantly higher than other areas. This was not consistent with our hypothesis that the diversity of healthy sand was higher than degraded sand. Guo et al. [43] studied the ectomycorrhizal community structure of *P. sylvestris* at different scales and showed the same results, i.e., that the level of diversity was not correlated with the health status of the stand. Microbial diversity is mainly influenced by tree species [48,49]; no definitive study has shown that the diversity of healthy vegetation is higher. There was no significant difference in the evenness index except for the bacterial community in HB Sandy Land and HQ Sandy Land, indicating that the distribution of bacterial community was relatively uniform in all locations.

### 4.2. Variation in Bacterial and Fungal Community Structure

We found that Actinobacteria, Proteobacteria, Chloroflexi and Acidobacteria were the dominant taxonomic phyla in the bacterial community composition—a finding in accordance with those of Zhao [50] and Kang et al. [51]. Actinobacteria, involved in the complex of soil organic matter decomposition [52,53], such as cellulose and lignin, which explains why the relative abundance was highest in all locations. In addition, it also possesses a strong tolerance to drought and can produce substances that stimulate and promote plant growth [54]. Proteobacteria are considered eutrophic, with higher relative abundance in soils with high available carbon content [55], which is consistent with the result that the relative abundance of Proteobacteria were HB (25.83%) > OD (20.83%) > HQ (20.20%) > MU (17.33%). However, in other studies on the soil bacteria of *P. sylvestris*, Chloroflexi does not belong to the dominant phylum [42,56]. Previous studies showed that Chloroflexi also participates in the cycle of carbon and nitrogen except, for fixing CO_2_ [57]. Acidobacteria is a group of acidophilic bacteria, which also effect the degradation of cellulose [58]. Pankratov et al. [59] found that, although the degradation function of Acidobacteria was not as good as other known cellulose-degrading bacteria, it possessed strong stress resistance and could survive in cold northern acidic wetlands, playing an important role in cellulose degradation under such conditions. 

As far as fungi are concerned, Ascomycota and Basidiomycota were the main dominant groups, which has been confirmed in many studies [60,61,62]. The relative abundance of Basidiomycota was higher than that of Ascomycota in all samples collected. Ascomycota are dominated by saprophytic fungi, which are involved in the decomposition of organic matter and nutrient cycling [63,64]. Basidiomycota can decompose lignocellulose and cause symbiosis with plants to form mycorrhiza [9,61,65]. Although the Ascomycota and Basidiomycota are the dominant phyla in the MU Sandy Land, the relative abundance of the Ascomycota (56.07%) in the MU Sandy Land is greater than that of the Basidiomycota (38.46%). Ascomycota was able to decompose complex organic matter and held a faster evolution rate, stronger drought tolerance and radiation tolerance, and, thus, is more suitable for survival in harsh environments [62].

To sum up, the relative abundance of bacterial and fungal taxa in soil depends on the spatial variation of soil environment and specific characteristics of sampling sites [66].

### 4.3. Response of Bacterial and Fungal Communities to Environmental Variation

The significant differences in the microbial community structure of *P. sylvestris* from the original locality to the introduced area resulted from the environmental heterogeneity of climate and soil factors. According to the Mantel test, meteorological factors have significant effects on bacterial and fungal community structures at large scale. Prior studies noted that soil properties, precipitation, and temperature constitute the key drives of soil microbial composition and metabolic potentials at large ecological scales [67,68,69]. In an Alaskan boreal forest, the response of microbial communities to warming was decreasing richness [70]. Zhou et al. [71] investigated the effect of temperature on the formation of microbial diversity at various scales and found that temperature showed a regulatory effect on microbial community diversity and microbial activities. In terrestrial environments, metabolic rates and ecosystem productivity generally increase as temperatures rise [72,73,74,75]. Ten years of irrigation in a water-limited pine forest increased tree growth and canopy cover, as well as litter fall and root biomass, and microbial communities that thrived as a result of the increased availability of resources [76]. Bacteria and fungi communities showed different adaptability to precipitation changes. Fungi showed stronger tolerance to water stress due to the existence of mycelia [77,78]. The response of microorganisms to temperature and precipitation at different ecological scales still needs further comprehensive research. The high heterogeneity of soil granular structure, porosity, water holding capacity and other aspects provides living space, sufficient nutrient conditions and a suitable microenvironment for various microorganisms, which is the basis of rich soil microbial diversity [79].

In this study, Mantel test results showed that the pH was closely related to both the soil bacterial and fungal community compositions. It has been suggested that pH is a major regulator of microbial diversity and community composition at the regional scale [80]. Soil pH regulates microbial growth associated with plant nutrients availability by controlling the morphology of compounds in the soil. Zhang et al. [81] proved that soil pH could change the microbial utilization efficiency of nutrients, physiological metabolic activity and competition between populations, directly or indirectly affecting microbial diversity. In addition, Mantel tests revealed that the total nitrogen and soil organic carbon content was a major factor influencing the bacterial community structure. Decomposition of litter, roots and dead biological cells is the main source of forest soil organic carbon, which is an important part of the carbon cycle [82]. Sul et al. [83] found that soil microbial community structure and specific group distribution were most affected by soil organic carbon. SOC was also shown to mediate the geographic distribution of the microbial communities in complicated and volatile environmental situations [84,85,86]. Total nitrogen content was the main factor affecting the EM fungal community composition [43]. The diversity and composition of microbial communities vary greatly at the spatial scale; the complex interaction mechanism among soil environment, meteorological factors and plants still needs to be further explored. In addition, we only analyzed the differences of community composition in a single period; thus, further long-term studies on the seasonal dynamics of microorganisms are needed.

## 5. Conclusions

In this study, the response of microorganisms to environmental changes on a large spatial scale was studied. The results showed that there were obvious regional differences in bacterial and fungal community composition and different sensitivities to environmental factors. Microbial community structure showed a close correlation with meteorological factors; pH had a significant effect on the microbial community. In addition, bacterial community was sensitive to the content of N and C. Contrary to our expectations, the microbial α diversity in healthy stands was not higher than that in declining stands; further study is needed to explore this phenomenon. 

The degree of microbial community response to the change of environmental factors varies in different sandy lands. Understanding this correlational analysis could provide theoretical support to reduce the decline of *P. sylvestris* forest in the introduced areas.

## Figures and Tables

**Table 1:** General information of sampling sites.

Site	HB	OD	MU	HQ
GC	48°14′–48°18′ N 119°58′–120°1′ E	42°22′–42°24′ N 117°15′–117°17′ E	38°18′–38°22′ N 109°40′–109°46′ E	42°42′–42°43′ N 122°29′–122°30′ E
Ele (m)	790–850	1520–1530	1100–1130	220–240
Ta (°C)	11.6	14.7	11.0	9.9
T+ (mm)	18.4	21.9	18.6	16.6
T− (mm)	4.4	7.6	3.9	3.5
Pa (mm)	22.7	18.0	18.7	24.8
Soil type	aeolian sandy soil	aeolian sandy soil	aeolian sandy soil	aeolian sandy soil
Stand status	normal growth	normal growth	part of the degradation	part of the degradation

GC: geographic coordinates; Ele: elevation; Ta: the mean monthly temperature; T+: the monthly maximum temperature; T−: the monthly minimum temperature; Pa: monthly maximum temperture. HB: the Hulunbuir Sandy Land; HQ: the Horqin Sandy Land; MU: the Mu Us Sandy Land; OD: the Otindag Sandy Land.

**Table 2:** Soil properties compared by ANOVA (mean values ± S.E.).

Site	TN	SOC	AP	pH	CEC
HB	0.11 ± 0.01 a	1.35 ± 0.11 a	8.61 ± 1.05 a	5.88 ± 0.02 c	8.66 ± 0.72 a
HQ	0.04 ± 0.00 b	0.42 ± 0.04 b	2.78 ± 0.25 c	6.29 ± 0.11 b	2.65 ± 0.13 c
MU	0.01 ± 0.01 c	0.18 ± 0.01 c	4.32 ± 0.18 bc	8.31 ± 0.04 a	2.88 ± 0.06 c
OD	0.10 ± 0.01 a	1.39 ± 0.08 a	5.39± 0.24 b	6.15 ± 0.02 b	5.35 ± 0.33 b

TN: total nitrogen; SOC: soil organic carbon; AP: available phosphorus; pH: pH value; CEC: cation exchange capacity. Different letters indicate significant differences among different sandy land tested by one-way ANOVA (*p* < 0.05).

**Table 3:** Spearman correlation coefficients for bacterial diversity and environmental variables (* *p* < 0.05; ** *p* < 0.01).

	TN	SOC	AP	pH	CEC	Pa	T−	Ta	T+
Shannon	−0.261 **	−0.287 **	−0.385 **	0.344 **	−0.19 *	0.105	−0.246 **	−0.246 **	−0.105
Chao1	−0.306 **	−0.312 **	−0.226 *	0.353 **	−0.159	−0.026	−0.134	−0.134	0.026
Pielou	0.016	−0.025	−0.313 **	0.181 *	−0.05	0.055	−0.133	−0.133	−0.055

**Table 4:** Spearman correlation coefficients for fungal diversity and environmental variables. (* *p* < 0.05; ** *p* < 0.01).

	TN	SOC	AP	pH	CEC	Pa	T−	Ta	T+
Shannon	−0.160	−0.166	−0.257 **	0.201 *	−0.215 *	0.015	−0.162	−0.162	−0.015
Chao1	−0.132	−0.131	−0.177	0.196 *	−0.152	−0.075	−0.066	−0.066	0.075
Pielou	−0.211 *	−0.238 **	−0.484 **	0.126	−0.363 **	0.372 **	−0.519 **	−0.519 **	−0.372 **

**Table 5:** Correlations between bacterial community composition and climate factors/soil properties assessed by Mantel tests.

	TN	SOC	AP	pH	CEC	Pa	T−	Ta	T+
r	0.15346	0.16540	0.00052	0.07494	0.03636	0.32330	0.36799	0.44175	0.47109
*p*	0.002	0.001	0.465	0.042	0.209	0.001	0.001	0.001	0.001

**Table 6:** Correlations between fungal community composition and climate factors/soil properties as-sessed by Mantel tests.

	TN	SOC	AP	pH	CEC	Pa	T−	Ta	T+
r	0.0344	0.02711	0.01592	0.08055	0.04986	0.14459	0.14554	0.17623	0.19962
*p*	0.157	0.205	0.364	0.018	0.138	0.001	0.002	0.001	0.001

## Data Availability

The data and results of this study are available upon reasonable request. Please contact the main author of this publication.
